# Cardiomyopathy Induced by Artificial Cardiac Pacing: To Whom, When, Why, and How? Insights on Heart Failure Development

**DOI:** 10.21470/1678-9741-2021-0629

**Published:** 2023

**Authors:** Andres Di Leoni Ferrari, Eduardo Bartholomay Oliveira, Ana Paula Tagliari, Adriano Nunes Kochi, Thaís Mariel Andara Beuren, Gustavo Chiari Cabral, Flávio Vinicius Costa Ferreira, Luiz Cláudio Danzmann

**Affiliations:** 1 Cardiac Stimulation Unit, Hospital São Lucas, Pontifícia Universidade Católica do Rio Grande do Sul (PUCRS), Porto Alegre, Rio Grande do Sul, Brazil; 2 Department of Electrophysiology, Hospital São Lucas, Pontifícia Universidade Católica do Rio Grande do Sul (PUCRS), Porto Alegre, Rio Grande do Sul, Brazil; 3 Cardiovascular Surgery Service, Hospital São Lucas, Pontifícia Universidade Católica do Rio Grande do Sul (PUCRS), Porto Alegre, Rio Grande do Sul, Brazil; 4 Cardiology Service, Hospital São Lucas, Pontifícia Universidade Católica do Rio Grande do Sul (PUCRS), Porto Alegre, Rio Grande do Sul, Brazil; 5 Heart Failure Unit, Hospital São Lucas, Pontifícia Universidade Católica do Rio Grande do Sul (PUCRS), Porto Alegre, Rio Grande do Sul, Brazil

**Keywords:** Heart Failure, Cardiac Pacing, Artificial, Cardiomyopathies, Ventricular Function, Biological Factors.

## Abstract

Coordinated and harmonic (synchronous) ventricular electrical activation is essential for better left ventricular systolic function. Intraventricular conduction abnormalities, such as left bundle branch block due to artificial cardiac pacing, lead to electromechanical “dyssynchronopathy” with deleterious structural and clinical consequences. The aim of this review was to describe and improve the understanding of all the processes connecting the several mechanisms involved in the development of artificially induced ventricular dyssynchrony by cardiac pacing, most known as pacing-induced cardiomyopathy (PiCM). The chronic effect of abnormal impulse conduction and nonphysiological ectopic activation by artificial cardiac pacing is suspected to affect metabolism and myocardial perfusion, triggering regional differences in the activation/contraction processes that cause electrical and structural remodeling due to damage, inflammation, and fibrosis of the cardiac tissue. The effect of artificial cardiac pacing on ventricular function and structure can be multifactorial, and biological factors underlying PiCM could affect the time and probability of developing the condition. PiCM has not been included in the traditional classification of cardiomyopathies, which can hinder detection. This article reviews the available evidence for pacing-induced cardiovascular disease, the current understanding of its pathophysiology, and reinforces the adverse effects of right ventricular pacing, especially right ventricular pacing burden (commonly measured in percentage) and its repercussion on ventricular contraction (reflected by the impact on left ventricular systolic function). These effects might be the main defining criteria and determining mechanisms of the pathophysiology and the clinical repercussion seen on patients.

## INTRODUCTION

Permanent pacemaker (PPM) implantation is the best therapeutic choice for symptomatic bradyarrhythmias^[^1^,^2^]^. However, artificial cardiac pacing (ACP), especially right ventricular (RV) apical pacing, also known as “conventional” ACP, may induce inter and intraventricular dyssynchrony, increase sympathetic activation, cause abnormalities in myocardial perfusion and endothelial function, and worsen cardiac output, resulting in poor cardiovascular outcomes^[^2^]^. Although ACP is an effective therapy for heart rhythm disorders that restores heart rate and cardiac hemodynamics, it may induce ventricular dyssynchrony, which is clinically manifested as heart failure (HF) and/or arrhythmia (atrial fibrillation [AF])^[^3^]^. This process results from an artificially induced ventricular dyssynchrony caused by cardiac pacing or, in a broader pathophysiological concept, pacing-induced cardiomyopathy (PiCM).

Taking this into consideration, this article aims to provide an update of the current evidence for PiCM by focusing on new issues and advances in the field. Definitions, pathophysiology, predictors, and the continuing challenge of RV pacing will be discussed. For this purpose, we conducted a literature review on the topic to familiarize cardiovascular surgeons, cardiologists, and other experienced practitioners working with this well-characterized condition^[^1^,^4^-^7^]^. We searched PubMed®, Cochrane, Medscape, and Ovid® databases for articles published since 2007 using the following terms: “cardiomyopathy”, “heart failure”, and “permanent pacemaker.”

## DEFINITION OF PiCM AND EVIDENCE FOR ADVERSE EFFECTS OF ACP

Among possible ACP sites, the RV apex has been the traditional choice. The RV apex is easily accessible anatomically and provides sufficient electronic stability and reliability for lead implantation^[^1^,^2^]^. Although most patients undergoing ACP remain clinically stable for years and have a good quality of life after the procedure, artificial electrical activation of the myocardium, or artificially induced ventricular dyssynchrony due to cardiac pacing, has been associated with the development of left ventricular (LV) dilation, worsening left ventricular ejection fraction (LVEF), arrhythmia, and clinical manifestations of HF^[^1^,^4^,^8^,^9^]^.

Importantly, despite the term “pacing-induced cardiomyopathy” being widely used and acknowledged by cardiologists, it has not been supported by the European Society of Cardiology’s^[^10^]^ nor the American Heart Association’s^[^11^]^ definitions of cardiomyopathies. This could be in part because research over the last decades has led to an increasing appraisal of previously unknown adverse effects associated with long-term RV pacing. For instance, dilated cardiomyopathy is characterized by the presence of LV or biventricular dilatation and systolic dysfunction without abnormal loading conditions, or coronary artery disease sufficient to cause global systolic impairment^[^12^]^. In this context, the concept of “pacing-induced heart disease” triggered by ACP-induced dyssynchrony has gained recognition.

Therefore, PiCM can be defined as a significant decrease in LVEF in patients with high percentages of RV pacing when other potential causes have been ruled out^[^13^,^14^]^. In patients with complete atrioventricular block (AVB) and pre-implantation LVEF > 50%, RV PiCM is defined as a subsequent need for cardiac resynchronization therapy (CRT) upgrade or a decrease in LVEF to ≤ 40% after PPM implantation^[^15^]^. According to Kaye et al.^[^16^]^, PiCM may be defined according to three different definitions:

- **Definition 1**: LVEF decrease to ≤ 40% in patients with previous LVEF ≥ 50%, or an absolute LVEF ≥ 5% reduction in patients with baseline LVEF < 50%. This definition is based on the fact that LVEF ≤ 40% would probably have a clinical impact on medical therapy or CRT indication.
- **Definition 2**: LVEF decrease to ≤ 40% in patients with previous LVEF ≥ 50%, or a 10% absolute reduction in patients with baseline LVEF < 50%. This definition is based on the fact that an absolute reduction in LVEF ≥ 10% would be more clinically relevant than an absolute reduction in LVEF ≥ 5%^[^16^]^.
- **Definition 3**: a reduction in LVEF ≥ 10% irrespective of baseline LVEF. This definition is relevant as it has been used in previous publications^[^17^]^.

Based on the broader definition (LVEF decrease to ≤ 40% or CRT upgrade), 12.3% of patients developed PiCM during a mean follow-up of 4.3 years in a cohort of 823 patients with normal baseline LVEF (> 50%) undergoing PPM implantation for third-degree AVB^[^14^]^. When the other definitions were considered, PiCM incidences ranging from 5.9% to 39% were reported: 9.3% according to definition 1; 5.9% according to definition 2; and 39.0% according to definition 3^[^16^]^. In multivariate analysis, the only independent factor associated with the development of cardiomyopathy was ventricular pacing burden (*P*=0.013)^[^16^]^. Based on these findings, the authors recommend that patients should have a baseline echocardiogram; the test should be repeated annually for patients with reduced LVEF (< 50%) and high rates of RV pacing (≥ 40%) and every two years for patients with preserved LVEF^[^9^]^. In a study in which PiCM was defined as a decrease in LVEF ≥ 10% resulting in LVEF < 50%, 19.5% of patients developed PiCM during a mean follow-up of 3.3 years^[^18^]^. Another study, in which PiCM was defined as a decrease in LVEF ≥ 10% with HF symptoms, reported an incidence of 20.5% during a mean follow-up of 15.6 years^[^19^]^.

Regarding time of onset of symptomatic artificially induced ventricular dyssynchrony by cardiac pacing, the reported mean time between PPM implantation and the first RV pacing-related HF event in patients with previously normal LVEF is two to five years^[^20^-^22^]^. Conversely, studies with implantable defibrillators that included patients with preexisting systolic dysfunction reported accelerated adverse responses to RV apical stimulation, resulting in overt HF after one year^[^23^]^. A PiCM incidence of 9% one year after PPM implantation and of 15.4% at the end of follow-up (15 years) was reported in a study using a rather different definition for diagnosis: LVEF ≤ 45%, LV dyskinesia in patients with complete AVB, and absence of other causes of cardiomyopathy (*e.g.*, cardiotoxic drugs and coronary artery disease)^[^4^]^.

Together, these findings suggest that one in every five patients with normal RV systolic function could have significantly decreased LVEF between one and four years after high rates of RV pacing. In many of these patients, RV pacing will also trigger clinical symptoms of HF and significantly increase the incidence of hospitalization for HF^[^24^]^.

## PATHOPHYSIOLOGY OF PiCM

Optimal cardiac performance demands vigorous systolic contractions and rapid diastolic relaxation. These events are sequential, precisely timed, and interdependent, requiring the rapid synchronous electrical stimulation provided by the His-Purkinje system. RV pacing generates slow asynchronous electrical stimulation, which disrupts the timing of the cardiac cycle and causes LV mechanical asynchrony^[^25^]^.

The electromechanical myocardial dyssynchrony caused by RV pacing is suspected to have adverse effects on ventricular function, inducing structural remodeling due to changes in myocardial metabolism and perfusion^[^24^,^26^-^28^]^. The true incidence of ventricular remodeling following artificially induced ventricular dyssynchrony due to RV pacing remains unknown, but it has been widely acknowledged to occur mostly in patients with RV pacing burden > 40%^[^29^,^30^]^. This cutoff point was recently reviewed in the current European Society of Cardiology Guidelines^[^31^]^, which acknowledged that changes caused by RV pacing can occur in patients with RV pacing ≥ 20%^[^12^,^31^]^. However, surprisingly and for reasons still unclear, most patients with pacing burdens close to 100% do not develop LV dysfunction or remodeling^[^32^]^.

“Conventional” RV pacing (apical) causes myocardial activation almost in reverse to that which occurs in intrinsic physiological antegrade conduction using the His-Purkinje system. It causes delayed and abnormal ventricular myocardial activation (“electrical” dyssynchrony) associated with nonphysiological ventricular contraction (“mechanical” dyssynchrony)^[^5^]^, indicating that RV pacing causes the dyssynchrony that leads to PiCM. Interventricular dyssynchrony (between the right and the left ventricles) results from the delay between electrical activation at the RV pacing site and activation of the LV posterolateral wall, whereas intraventricular dyssynchrony results from the delay between regional activation within the ventricle^[^33^]^. Mechanical and electrical dyssynchrony results in prolongation of the systolic phase and shortening of the diastolic phase, compromising cardiac output, reducing diastolic ventricular filling, and causing functional mitral regurgitation^[^5^]^. Other structural changes include left atrial and LV remodeling, LV wall thickening, and cellular and intracellular changes (*e.g.*, degenerative fibrosis)^[^9^]^ attributed to the “inflammatory” (and toxic?) process triggered by ectopic electrical activation of the myocyte^[^34^]^. Chronic RV pacing has also been associated with increased sympathetic activation^[^35^,^36^]^, as well as increased oxidative stress associated with reduced nitric oxide production in the myocytes^[^37^]^.

Potential long-term adverse effects of RV pacing can occur in patients with both preserved and reduced LVEF, although they are more prominent in the latter. Data of paramount importance from the Mode Selection Trial (MOST) analyzing patients with sinus node dysfunction (SND), which randomly compared 707 patients with dual-chamber rate-modulated (DDDR) pacing *vs.* 632 with single-chamber ventricular rate-modulated (VVIR) pacing^[^29^]^, show that after a mean follow-up of 33.1 months, the risk of hospitalizations for HF and AF was directly correlated with RV pacing burden, irrespective of pacing mode (single- or dual-chamber). When adjusted for baseline clinical covariates, ventricular pacing > 40% was associated with a 2.9 increase in HF hospitalization and a 1.36 increase in the risk of AF. For single-chamber pacing, these numbers were similarly alarming: pacing > 80% was associated with a 2.56 increase in HF hospitalization, and the risk of AF increased 1.21 times with every 25% increase in RV pacing burden^[^29^]^. However, furthering the debate, although widely cited, the study findings are not entirely elucidated. DDDR (a theoretically more physiological pacing mode for respecting AV synchrony) required half the RV pacing burden to trigger pacing-related adverse effects compared to VVIR (40% *vs.* 80%, respectively)^[^29^]^. In addition, a subanalysis of MOST revealed that < 10% of patients actually developed HF, primarily those with coronary artery disease or previous structural heart disease^[^38^]^.

In the Protection of Left Ventricular Function During Right Ventricular Pacing (or PROTECT-PACE), an interesting trial focused on elucidating the importance of RV pacing site, 240 patients with high grade AVB requiring > 90% ventricular pacing and preserved LVEF > 50% were randomly assigned to receive RV apical pacing (n=20) *vs.* high septal pacing (n=120). At two years of follow-up, LVEF decreased in both the apical (57±9 to 55±9%; *P*=0.047) and the high septal groups (56±10 to 54±10%; *P*=0.0003). There was no significant difference between sites (*P*=0.43). There was also no significant difference between the rates of HF hospitalization, mortality, AF, and natriuretic peptide levels between the two groups^[^39^]^.

A subanalysis of the Multicenter Automated Defibrillator Implantation Trial-II (MADIT-II) showed that patients with high RV pacing burden were at significant increased risk of new or worsened HF, supporting the deleterious effects of RV pacing distress^[^40^,^41^]^. Consequently, many PPM manufacturers introduced algorithms and several device-programming strategies to minimize unnecessary RV pacing. However, findings on the benefits of such strategies and algorithms are conflicting^[^42^]^. On one hand, a more recent meta-analysis of 10 randomized clinical trials with 6,639 patients designed to evaluate the effect of different algorithms on the risk of AF and HF in patients with SND found that the strategy to minimize ventricular pacing was associated with a reduction in the composite outcome of AF and HF (odds ratio [OR]: 0.66; *P*=0.007)^[^43^]^. On the other hand, a meta-analysis of seven randomized studies with 4,119 patients showed that algorithms may not have any specific benefits or superior clinical outcomes compared to standard DDDR programming in patients with normal LVEF^[^44^]^. There is abundant evidence for the deleterious effects of RV pacing, with strong data suggesting that RV pacing induces ventricular dyssynchrony and should, therefore, be avoided. A meta-analysis of seven randomized studies on RV pacing did not identify any impact on clinical outcomes, and the reasons might include the mean follow-up of 2.5 years (two out of seven studies only had a follow-up of one year), which is too short to detect any effect in patients with normal LVEF (the effects of dyssynchrony on ventricular function may take five years to become evident), and the sample of 2,000 patients with RV pacing prevention, which might be, in the field of cardiac pacing, too small to identify any effect on mortality^[^45^]^.

## PREDICTORS OF PiCM

**Fig. 1:**
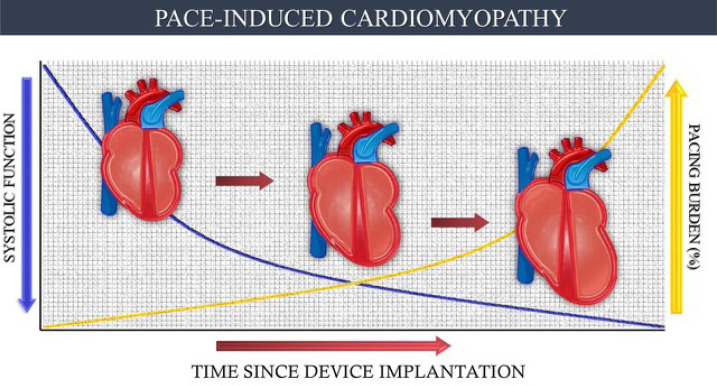
Cardiomyopathy induced by artificial cardiac pacing: pathophysiological evolution of artificially induced myocardial ventricular dyssynchrony.

The current relevance of PiCM is under debate, and its recognition relies on detailed diagnostic criteria and study population (Figure 1). In these cases, ventricular dilation must be clearly associated with the artificially induced ventricular dyssynchrony caused by ventricular stimulation, which is associated with electrical dyssynchrony (especially left bundle branch block [LBBB]) and not with the clinical and functional progression of the underlying heart disease (such as valvular, ischemic, or hypertensive cardiomyopathy). Efforts have been made to understand the pathophysiology and the development factors associated with PiCM after it was consistently reported by several publications^[^13^,^15^,^16^,^18^]^. RV PiCM is usually defined as LV systolic dysfunction resulting from electrical and mechanical dyssynchrony caused by RV pacing. RV PiCM is common and occurs in 10-20% of patients exposed to frequent RV pacing^[^46^]^. Multiple risk factors for PiCM have been identified, and the most investigated predictors are of clinical (HF), electrocardiographic (morphology and QRS duration), and echocardiographic (LVEF) natures^[^1^,^46^-^48^]^. However, a review of the available studies revealed a significant heterogeneity of results, given that PiCM has only recently been more deeply studied and acknowledged, and individualizing patients at higher risk is especially challenging in this context. Some of the reasons include: 1) there is no consensus among authors on the definition and pathophysiology of PiCM; 2) most studies are prospective, which means that the data is not yet consolidated and the results, as well as the inclusion criteria, are heterogeneous among them; 3) follow-up time varied greatly between studies, meaning that PiCM incidence is different if evaluated in distinct time frames. Therefore, any assumptions are invariably susceptible to erroneous conclusions due to the divergence between factors.

As with any other disease, the clinical objective of predictor analysis is to increase understanding of the disease and how to prevent it. In PiCM specifically, predictors are divided into two groups: pre- and post-implantation predictors. The presence of any of the currently recognized factors should lead to frequent clinical and echocardiographic follow-up in those at higher risk. Identifying pre-implantation predictors, such as native QRS duration, age, sex, underlying diseases, and expected RV pacing burden, among others, allows us to identify patients who may benefit from a more physiological ACP approach. However, this pacing mode has only been recently included in guidelines, after it was formally supported by clinical trials^[^30^]^.

Despite certain heterogeneity, a few variables were identified as predictors in more than one cohort, increasing reliability that these variables are true risk predictors - such is the case of RV pacing burden, discussed earlier. Among evaluated cohorts, RV pacing burden is listed a predictor in five of them (Table 1)^[^6^,^13^,^14^,^19^,^50^]^. Importantly, all five studies are observational, the majority of which is retrospective. This may weaken our conclusions because these particular characteristics are probably the most frequent limitations encountered when considering the relevance of the results. A retrospective analysis of 234 patients with a mean follow-up of 15.6 years reported a PiCM incidence of 20.5%. In multivariate analysis, predictors were age at implantation, longer paced QRS duration (when > 185 ms; sensitivity of 66.7% and specificity of 76.3%), a higher myocardial scar score on the electrocardiogram (Selvester QRS score), and a higher percentage of RV pacing^[^19^]^.

**Table 1:** Studies evaluating predictors of pacing-induced cardiomyopathy.

Study	Number of patients (N)	Study design	Mean follow-up (years)	Diagnostic criteria for pacing-induced cardiomyopathy	Predictors and overall outcomes
Khurshid et al.^[^15^]^	1,75	Retrospective cohort	3.3	Decrease in LVEF ≥ 10% + resultant LVEF < 50%	Male sex (HR: 2.15; 95% CI, 1.17-3.94; *P*=0.01) Native QRS duration (HR: 1.03; 95% CI, 1.01-1.05; *P*<0.001)
Lee et al.^[^16^]^	234	Retrospective cohort	15.6	Decrease in LVEF > 5% + HF symptoms	Advanced age (HR: 1.62; 95% CI, 1.22-2.16; *P*=0.001) Paced QRS duration (HR: 1.54; 95% CI, 1.15-2.05; *P*=0.003) Myocardial scar score (HR: 1.23; 95% CI, 1.03-1.49; *P*=0.003) RV pacing burden (HR: 1.31; 95% CI, 1.01-1.49; *P*=0.01)
Kiehl et al.^[^10^]^	823	Prospective cohort	4.3	Post-implantation LVEF ≤ 40% or CRT upgrade	Reduced baseline LVEF (HR: 1.047; 95% CI, 1.002-1.087; *P*=0.042) RV pacing burden (HR: 1.011; 95% CI, 1.002-1.02; *P*=0.021)
Ahmed et al.^[^49^]^	55	Prospective cohort	1	Decrease in LVEF ≥ 5% or LVEF < 45% after 1 year	Decreased GLS on TTE after 1 month (-16.4 vs. -12.6; *P*=0.022)
Hayashi et al.^[^50^]^	115	Retrospective cohort	8.9	Decrease in LVEF ≥ 10% + resultant LVEF < 50%	RV pacing burden (OR: 1.04; 95% CI, 1.01-1.09; *P*=0.04) Paced QRS notching in leads II/DIII/aVF (OR: 5.04; 95% CI, 1.59-19.6; *P*=0.005) QS of paced QRS in V1-V6 (OR: 3.56; 95% CI, 1.21-10.8; *P*=0.02)
Bansal et al.^[^11^]^	363	Prospective cohort	1.2	Decrease in LVEF ≥ 10%	Pacing burden > 60% (HR: 4.26; 95% CI, 1.59-11.41; *P*=0.004) Aortopulmonary ejection delay (HR: 3.15; 95% CI, 1.52-6.55; *P*=0.002)
Cho et al.^[^6^]^	1,418	Retrospective cohort	7.2	Decrease in LVEF ≥ 10% or resultant LVEF < 50%	Previous LBBB (OR: 4.22; 95% CI, 1.34-13.3; *P*=0.01) Paced QRS duration (+10 ms) (OR: 1.11; 95% CI, 1.01-1.21; *P*=0.03) Pacing burden (OR: 1.01; 95% CI, 1.00-1.02; *P*=0.02)
Safak et al.^[^51^]^	170	Retrospective cohort	2	LVEF decrease to ≤ 45% + dyskinesia during RV pacing	Increased pre-implantation LVEF (OR: 0.88; 95% CI, 0.8-0.9; *P*=0.006) PPM indication for SND (OR: 0.1; 95% CI, 0.03-0.9; *P*=0.004)

CI=confidence interval; CRT=cardiac resynchronization therapy; GLS=global longitudinal strain; HF=heart failure; HR=hazard ratio; LBBB=left bundle branch block; LVEF=left ventricular ejection fraction; OR=odds ratio; PPM=permanent pacemaker; RV=right ventricular; SND=sinus node dysfunction; TTE=transthoracic echocardiogram

Although the follow-up period in the previous cohort was the longest^[^19^]^, another cohort had the highest number of patients: 1,750 patients followed for a mean of 3.3 years^[^18^]^. In this study, 19.5% of patients developed PiCM on control echocardiogram after one year, showing a reduction in mean LVEF from 62.1% to 36.2%. Male sex and native QRS duration were among predictors. From the 115 ms cutoff for baseline QRS duration (90% specificity), every 1 ms increase would increase the risk of PiCM by 3%^[^18^]^. PiCM progression has been suggested to occur mainly due to RV pacing duration in patients with previous structural disease, meaning that this patient profile is more susceptible to the “damages” caused by artificial pacing-related dyssynchrony^[^4^]^.

A total of 55 patients who received PPM for second- or third-degree AVB were followed in a study analyzing different echocardiographic parameters, such as strain analysis^[^49^]^.One month after implantation, 15% of patients had a decrease in LVEF ≥ 5%. After one year, control echocardiogram found that 27% of patients had a decrease in LVEF ≥ 5%. Baseline global longitudinal strain (GLS) did not differ between patients with or without further deterioration of systolic function. However, GLS values on the echocardiogram one month after implantation were lower in those who went on to develop decreased LVEF (-13.3±1.2 *vs.* -16.4±0.6; *P*=0.044). A cutoff value of -14.5 on the receiver operating characteristic curve gave a sensitivity of 82% and a specificity of 75%^[^49^]^.

Given that several studies have observed a relationship between paced QRS duration and PiCM development, it was hypothesized that electrode implantation at the RV apex could be a risk factor for cardiomyopathy due to its wider paced QRS compared to septal implantation. This hypothesis was tested in a study with 363 patients, of which 57.8% were assigned to receive apical pacing and 39.7% were assigned to septal pacing^[^14^]^. Indeed, QRS at nonapical pacing sites was significantly narrower than at apical pacing sites (139.7±17.7ms *vs.* 149.3±18.1ms; *P*<0.001). However, in multivariate analysis, apical pacing *per se* was not a predictor of PiCM (hazard ratio: 1.44; 95% confidence interval [CI], 0.66-3.14; *P*=0.355), whereas RV pacing burden > 60% and aortopulmonary ejection delay (echocardiogram marker of dyssynchrony) were identified as predictors of PiCM^[^14^]^.

The expected RV pacing burden differs according to each indication for PPM implantation. When comparing the incidence of PiCM between patients with SND *vs.* complete AVB, correlated with RV pacing percentage and paced QRS duration, there were no differences in HF admission between groups, although the complete AVB group had a higher pacing percentage, as expected^[^17^]^. Paced QRS duration ≥ 163 ms was the most important predictor of HF admission^[^17^]^.

## RV PiCM PATHOBIOLOGY: IS THERE A ROLE FOR BLOOD BIOMARKERS?

New perspectives on PiCM as a trigger of HF have emerged. Given that patients with symptomatic bradycardia will still require some form of pacing for the next several years, strategies for early detection, prevention, and treatment of people at risk for PiCM are of outmost importance. In fact, the range of molecules with diagnostic, prognostic, and therapeutic management potential related to HF has been of great interest in recent years. Natriuretic peptides and other biomarkers have already been validated and established, being currently a grade I recommendation in HF guidelines^[^2^,^43^]^ for providing valuable information for HF diagnosis. Taking into consideration these and other molecules that may appear in the near future, the hypothesis of their usefulness as risk markers for clinical development and tools for prognostic prediction and stratification in PiCM is raised.

The identification of serum biomarkers represents advances in precision medicine as potential therapeutic targets. Different levels of cardiac dyssynchrony primers are caused by ACP - in addition to LV dysfunction and remodeling^[^25^]^, RV pacing triggers a chronic process of electrical toxicity that could lead to tissue damage, inflammation, and myocardial fibrosis, combined with the mechanism of arrhythmia promoted by nonphysiological ectopic contraction. It has been acknowledged that several patients have a long pre-clinical phase characterized by few (if any) symptoms and minor cardiac abnormalities that deviate from current disease definitions^[^12^]^.

The movement from symptom to treatments that target specific disease mechanisms (especially in cardiac pacing and HF) is a conceptual shift from slowing disease progression to a pattern of reversal or prevention as the main objective. New biomarkers functioning as biotechnological tools that measure key cardiovascular variables could potentially generate data that might shed light on cardiac disruptions (*e.g.*, muscular misalignment and metabolic myocyte disturbances) and help to identify subsets of patients who are more vulnerable to PiCM. This could allow better quantification of cardiac dyssynchrony progression and lead to new interventions that could be used in the clinical setting and improve risk prediction, screening, and therapeutic monitoring^[^52^]^.

In daily practice, decisions regarding patient management are based on readily available clinical parameters and values obtained from various diagnostic techniques with prognostic relevance. Despite gaps in knowledge, precision medicine is no longer a theoretical model, but rather a real opportunity for the future treatment of patients with PiCM. In the near future, the identification of likely pathogenic variants that clinically manifest as HF in this setting might be validated and should be promoted to apply individualized therapeutic strategies^[^12^]^. A novel approach to patients with PiCM consisting of clinical parameters and biomarkers, as well as several easily obtainable structural measures (LVEF, LV mass, RV function etc.), should provide comprehensive information to guide patient-care diagnostic decisions and management strategies^[^53^]^.

## FROM PATHOPHYSIOLOGY TO PREVENTION USING PACING STRATEGIES

It is well known that prevention is the best approach to any disease, and such is the case with PiCM. Therefore, in addition to careful patient selection for PPM implantation, the individualized choice of device and programming are essential initial measures to reduce PiCM incidence. Surprisingly, 10% to 30% of patients in the Dual Chamber and VVI Implantable Defibrillator (DAVID) trial received unnecessary ventricular pacing^[^54^]^. This might have happened because the defibrillator devices used in the trial did not provide automatic prolongation of the atrioventricular (AV) interval that could result in efficient avoidance of RV pacing; in addition, the variability in intrinsic AV conduction at higher rates could be analyzed by the authors^[^54^]^. Ventricular pacing burden (as widely discussed above) is one of the factors with the greatest impact on PiCM development, meaning that higher percentages of RV pacing by PPM increase the likelihood of developing HF. A cutoff value of > 40% has been demonstrated in several studies^[^55^]^. For DDDR pacing, proper programming of AV intervals to allow intrinsic conduction can be achieved with a simple interval prolongation or by using device algorithms intended for this purpose^[^56^,^57^]^. As for VVIR pacing, programming the device at a lower heart rate than intrinsic, when possible, is the only way to try to reduce burden-related PiCM.

**Fig. 2:**
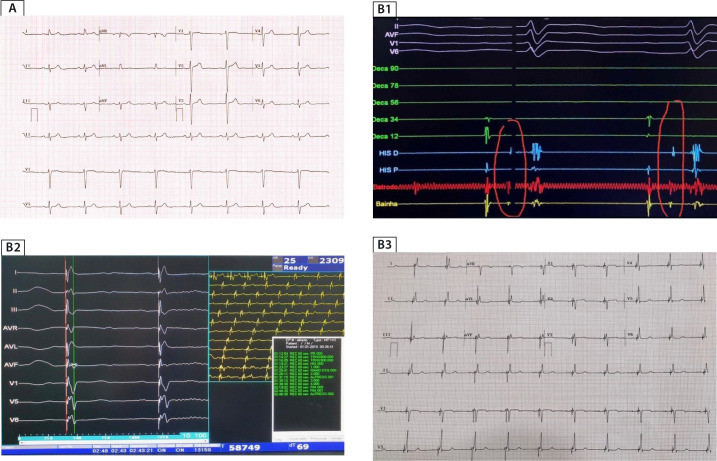
Physiological ventricular pacing on electrocardiogram. A) Baseline electrocardiogram with intrinsic QRS; B1) Signaling of His (100ms sweep speed) previous to lead deployment; B2) QRS duration during artificial pacing with bundle of His capture (Conduction System Pacing); B3) Final ECG with Conduction System Pacing and near intrinsic morphology of the paced QRS;

However, for patients requiring frequent RV pacing, the ventricular lead implantation site could be the only way to prevent PiCM. Ventricular contraction by direct artificial activation of the conduction system through His bundle capture is an elegant way to maintain ventricular syncytium synchrony. Physiological pathways are used in new physiological pacing strategies (PPS), preserving natural ventricular depolarization and preventing RV pacing-induced dyssynchrony (Figure 2A)^[^58^,^59^]^. For patients with AV node block below the His bundle and/or LBBB, for whom bundle pacing does not correct LV depolarization, left bundle branch pacing is an attractive alternative to His bundle pacing (HBP), with lower thresholds, a more attractive learning curve, and comparable results^[^60^]^. The deep septal approach allows direct left bundle branch pacing while preserving LV synchrony (Figure 2B)^[^61^]^.

These artificial activation strategies can be characterized as physiological ventricular pacing or, more precisely, PPS. Although conceptualized in the 1970s, they were not broadly developed until recently, possibly due to initial technical difficulties, and have been expanding in an attempt to conduct ACP as naturally as possible^[^62^]^. Initial studies have shown promising results for physiological ACP from reproducibility and safety perspectives; however, this technique is still limited to a few centers, thus most publications are case series and case-control studies and/or small randomized studies^[^63^-^65^]^.

A systematic review aiming to compile available data included randomized and observational studies comparing HBP or CRT *vs.* RV pacing in patients with LVEF > 35%^[^66^]^. Eight studies with 679 patients were included. After a follow-up of 1.57 years, patients who received RV pacing had significantly decreased LVEF, whereas patients who received CRT and HBP had a 5.3% absolute increase in LVEF compared to “conventional” RV pacing (95% CI, 2.86-7,8%; *P*<0.001). In the analysis limited to studies comparing HBP *vs.* RV pacing, HBP was associated with a 4.33% increase in LVEF (95% CI, 0.85-7.81%; *P*<0.01) during a mean follow-up of 8.36 months and an improvement in New York Heart Association (or NYHA) functional class (*P*=0.027) after a mean follow-up of 8.71 months. Patients with baseline LVEF < 50% and ventricular pacing > 40% benefited the most from HBP or CRT^[^66^]^. Afterwards, American guidelines included HBP as a grade IIa recommendation for patients with these specific characteristics^[^19^]^.

A case-control study compared results from two centers: one using HBP (successful in 304 of 332 patients [92%]) and another using RV pacing (successful in 137 of 433 patients [32%])^[^67^]^. The composite endpoint of death, HF hospitalization, or upgrade to CRT was 25% in the HBP group and 43% in the RV pacing group (OR: 0.71; 95% CI, 0.53-0.94; *P*=0.02). The most significant difference between pacing methods occurred primarily among patients from the RV pacing group with ventricular pacing burden > 20% (OR in this specific group: 0.65; 95% CI, 0.45-0.92; *P*=0.02).

Based on these results, can we state that PPS can prevent PiCM and should be routinely recommended at least on an individualized basis? It could be argued that the success of physiological pacing is in part related to the use of electrophysiological parameters when performing this technique, which allows proper identification of the target region. Although this procedure is more complex and implies a potential rise in costs, we propose this debate because we believe that PiCM is secondary to artificially induced myocardial dyssynchrony by RV pacing. Why would a patient with preserved physiological pacing of the ventricular syncytium (Figure 2A) develop PiCM? Importantly, not even CRT, which performs ACP via two wavefronts (right ventricle + left ventricle; the latter being epicardial), stimulates the ventricular syncytium as physiologically as PPS.

However, although CRT was shown to decrease total and cardiovascular mortality, among other HF-related outcomes, in patients with ejection fraction < 35% and QRS > 120 ms due to LBBB^[^68^]^, it was also shown to reduce PiCM incidence in the context of ACP. On the Biventricular *versus* Right Ventricular Pacing in Heart Failure Patients with Atrioventricular Block (or BLOCK-HF) study, 691 patients with AVB and LVEF < 50% were randomized to receive CRT *vs.* RV pacing by PPM^[^69^]^. After a mean follow-up of 37 months, the CRT group had a 26% reduction (OR: 0.74; 95% CI, 0.60-0.90) in the combined endpoint of HF hospitalization requiring intravenous medication and a 15% increase in LV end-systolic volume.

In the context of AF, the Left Ventricular-Based Cardiac Stimulation Post AV Nodal Ablation Evaluation (or PAVE) study randomized 184 patients undergoing ablation of the AV node to receive CRT *vs.* RV pacing by PPM^[^70^]^. Compared to the RV pacing group, the CRT group had improved six-minute walk distance (24% *vs.* 31%, respectively; *P*=0.04) and LVEF (0.41% *vs.* 0.45%, respectively; *P*=0.03) after a mean follow-up of six months. Similar findings were observed in a subgroup of patients undergoing ablation of the AV node and CRT or HBP when compared to RV pacing^[^66^]^.

However, in addition to current studies, further robust clinical trials comparing RV pacing *vs.* PPS are needed to consolidate HBP as an initial strategy for reducing PiCM. Several factors should be considered, including implant-related complications such as higher capture thresholds and undersensing and tool costs (dedicated leads and sheaths). In addition, some post-implantation factors are of great influence, such as increased battery depletion and premature generator replacement (due to higher energy drain), among others. These aspects need to be tested in a randomized, controlled setting before PPS can be widely used. There are currently several clinical trials comparing PPS *vs.* RV pacing and, more ambitiously, PPS *vs.* CRT. The results will demonstrate the true value of physiological artificial activation within the context of ACP and the possibility of significantly reducing PiCM incidence.

## CONCLUSION

Artificially induced myocardial dyssynchrony by RV pacing leading to PiCM is a recently acknowledged condition. Its potential genesis occurs after RV pacing initiation (single- or dual-chamber) and is probably associated with factors that are prone to this condition (underlying heart disease or phenotype). It is caused by artificial ventricular dyssynchrony due to artificial electrical cardiac activation of the myocardium only. Its impact on ventricular function and association with arrhythmia and congestive HF should be considered. Therefore, sentinel biomarkers could be used in the future to identify, prior to implantation, individuals who have a natural tendency to develop artificially induced dyssynchrony. In addition, strict clinical follow-up with evaluation of cardiac function and structure should be performed periodically (although there is still no established consensus on periodicity). Correction involves using biventricular cardiac pacing or the new physiological alternatives, via PPS (direct His bundle or left bundle activation), to minimize ventricular dyssynchrony.
